# Blinded two-phase evaluation of large language models in complex cardiac surgery: task-specific performance and human-AI collaboration

**DOI:** 10.3389/fdgth.2026.1769467

**Published:** 2026-05-29

**Authors:** Marc Leon, Ruibin Feng, Manuel Quiroz Flores, Glenn Pelletier, Daniel Bethencourt, Masafumi Shibata, Hao He, Chawannuch Ruaengsri

**Affiliations:** 1 Department of Cardiothoracic Surgery, Stanford University School of Medicine, Stanford, CA, United States; 2 Department of Medicine, Stanford University School of Medicine, Palo Alto, CA, United States

**Keywords:** blinded two-phase evaluation, cardiac surgery, clinical reasoning, human–AI collaboration, large language models

## Abstract

**Background:**

Large language models (LLMs) have demonstrated strong performance on standardized medical benchmarks. However, their potential in complex surgical decision-making is largely uncharacterized. Critically, human–LLM collaboration regarding the extent to which clinicians can effectively recognize and integrate model-generated reasoning has emerged as an unaddressed question. To address these gaps, we developed a two-phase evaluation framework to simultaneously assess LLM performance and human–LLM collaboration in cardiac surgery.

**Methods:**

A panel of senior cardiac surgeons independently developed 15 high-fidelity cardiac surgery scenarios, each paired with a clinically relevant open-ended reasoning task, expert-curated reference answers, and a 10-dimensional weighted evaluation framework. Five representative LLMs (O1, O3-mini-high, DeepSeek-R1, GPT-4, and Llama3-OpenBioLLM-70B) were prompted using a multi-agent strategy. A separate group of senior surgeons conducted a blinded two-phase evaluation to assess model performance and evaluator judgment shifts: in the first round, they rated LLMs independently; in the second, they were shown the reference answers and invited to revise their ratings, with changes being optional.

**Results:**

LLM performance varied across scenarios, but relative rankings remained stable. Median normalized scores were highest for O1 (0.896), followed by O3-mini-high (0.854), DeepSeek-R1 (0.792), GPT-4 (0.667), and Llama3-OpenBioLLM-70B (0.521). Across evaluation dimensions, scenario comprehension scored highest (0.920), while patient safety (0.507), hallucination avoidance (0.549), and clinical efficiency (0.597) were lowest across models. Second-round normalized scores declined for four LLMs, with 7.57% of ratings revised from affirmative to negative and only 2.59% from negative to affirmative. Among the five highest-weighted evaluation dimensions, 10.16% of second-round ratings were revised from affirmative to negative.

**Conclusions:**

Reasoning-optimized LLMs outperformed all other models. However, all models exhibited clinical limitations, including poor performance in core evaluation dimensions and scenarios requiring complex, longitudinal reasoning tasks. Overacceptance was the dominant collaboration imbalance, reflecting that clinicians over-accepted model reasoning that appears clinically sound yet is incorrect or potentially harmful. These findings suggest that these LLMs are not yet ready for safe use in complex surgical settings due to both performance limitations and human–LLM collaboration imbalance.

## 1 Introduction

Large language models (LLMs) are transforming healthcare by enhancing communication, improving diagnosis, and enabling personalized decision support (1–3). Early models such as GPT-3 performed below primary care physicians (4), but frontier LLMs have achieved diagnostic and triage accuracy comparable to, and sometimes surpassing, human physicians (5, 6). While their potential is clear, safe clinical integration requires rigorous validation and specialty-specific evaluation. Despite growing interest, several key gaps remain. First, most studies rely on standardized medical exams or simplified scenarios that assess general knowledge, rather than testing complex medical reasoning or nuanced decision-making, leaving reasoning-intensive domains underexplored (7). Second, although newer LLMs, particularly reasoning-optimized models, claim improved decision-making, their performance in complex clinical tasks remains untested. Third, prior studies have overwhelmingly focused on output accuracy (8), with limited attention to clinician-LLM interaction, specifically whether collaboration leads to underutilization of LLM output, or failure to detect seemingly reasonable but clinically significant errors. Understanding whether such interactions lead to appropriate trust calibration—or to systematic over- or under-reliance on AI-generated reasoning—is essential for safe clinical integration.

To address these gaps, this study focused on cardiac surgery, one of the most complex and underexplored specialties in LLM research. Unlike fields such as oncology or internal medicine, where LLMs benefit from structured guidelines and abundant training data (7), cardiac surgery involves high-stakes, time-sensitive decisions requiring integration of dynamic physiology, multidisciplinary data, long-chain reasoning, and highly specialized language rarely seen in general corpora (9, 10). This makes it an ideal setting to test clinical reasoning capabilities. We evaluated five representative LLMs, including reasoning-optimized, open-source, and a medical-tuned model, capturing a broad spectrum of current architectures and capabilities. We also introduced a novel two-phase evaluation framework to assess not only output quality but also how evaluator judgments evolve, enabling insight into human–LLM collaboration. Unlike prior benchmark- or vignette-based research, this is the first study to examine both LLM performance and human–LLM collaboration in complex, reasoning-intensive surgical scenarios, thereby bridging critical gaps in current LLM research.

## 2 Materials and methods

### 2.1 Study design and role assignment

**Figure 1:**
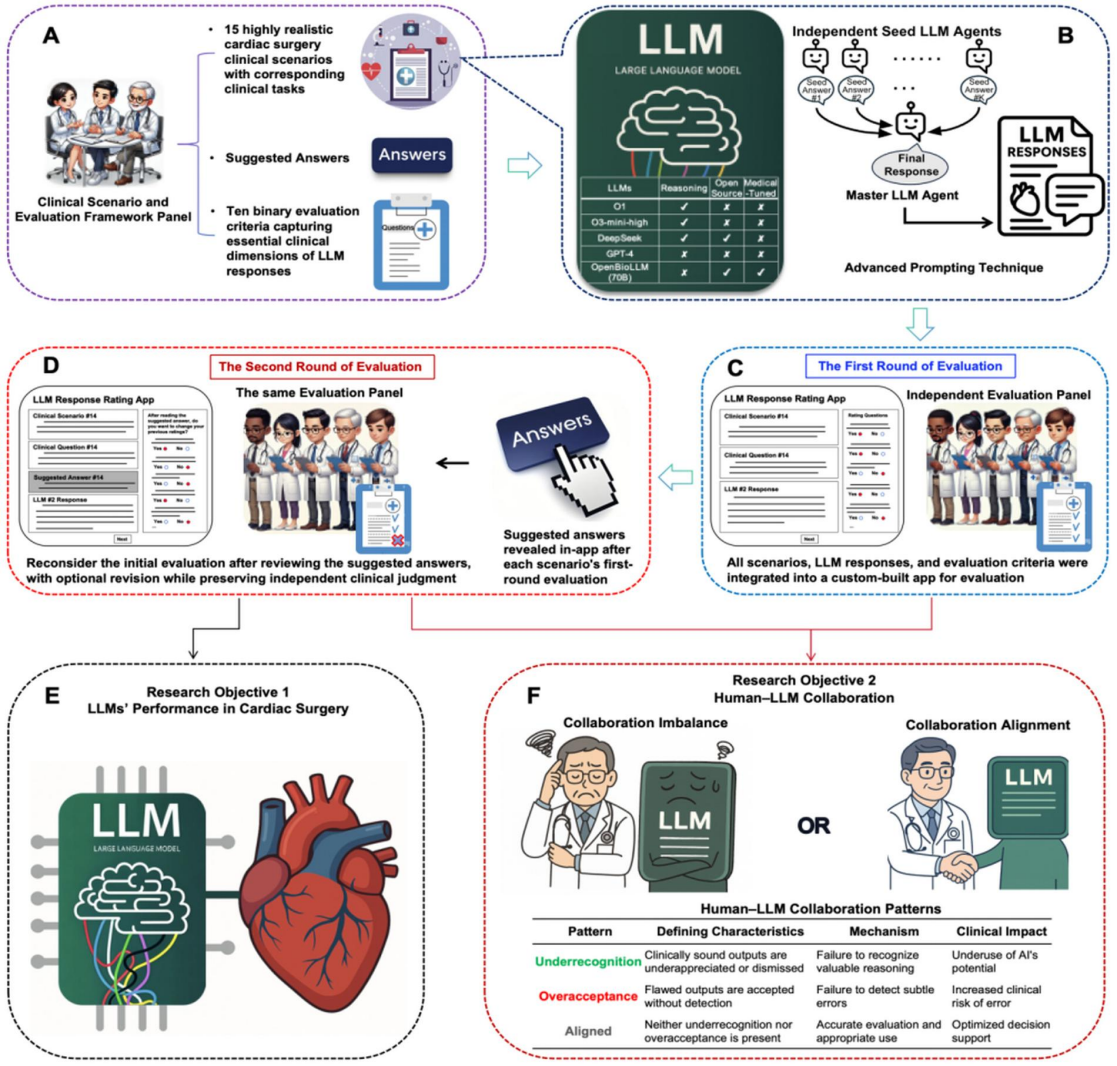
Study design, evaluation workflow, and human–LLM collaboration framework. **(A)**. panel of three board-certified senior cardiac surgeons developed 15 realistic clinical scenarios, corresponding reference answers, and ten binary criteria targeting key clinical competencies. **(B)**. LLM responses were generated using a multi-agent prompting strategy to improve output completeness and coherence while reducing variability. A master prompt synthesized three independent seed responses into a single final answer. **(C)**. In the first round, a separate panel of five board-certified senior cardiac surgeons independently rated LLM responses via a custom evaluation app. **(D)**. In the second round, the same evaluators reconsidered their initial ratings after reviewing the reference answers, with optional revisions while preserving independent clinical judgment. **(E)** Second-round ratings served as the primary basis for evaluating and comparing the clinical performance of the five LLMs. **(F)**. Differences between first- and second-round ratings were analyzed to characterize patterns of human–LLM collaboration.

Institutional review board approval and informed consent were waived, as the study involved no patient data or human subjects. Eight board-certified senior cardiac surgeons participated: three developed clinical scenarios and the evaluation framework, while five served as blinded evaluators. This study followed a two-phase, blinded evaluation design; a visual summary is provided in Figure 1.

### 2.2 Clinical scenarios and evaluation framework

Fifteen high-fidelity cardiac surgery scenarios were independently constructed by a panel of three senior cardiac surgeons, designed to elicit open-ended, free-text responses from the LLMs. The scenarios were inspired by real clinical encounters and informed by the panel's collective experience, relevant literature, and clinical guidelines, with appropriate modifications to ensure no identifiable patient data were included. This approach preserved clinical authenticity while avoiding potential model pre-exposure to published case reports. Tasks spanned multiple decision domains, including hemodynamic interpretation, pharmacologic or surgical planning, perioperative complication management, and advanced medical calculations. Each scenario included a task-specific clinical question and a reference answer. Detailed clinical scenarios, task-specific questions, reference answers, and model-generated responses are provided in Supplementary Data Sheets S1. LLM responses were evaluated using a ten-dimensional framework designed to comprehensively assess clinical performance. Each criterion was scored as Yes/No, with weighted scoring and normalization to a 0–1 scale. A *post-hoc* complexity classification was performed by the three scenario designers, who independently rated each scenario across five clinical dimensions (Supplementary Table S5); final scores represent the mean of the three independent ratings (Supplementary Table S6). The evaluation criteria, weighting scheme, and underlying rationale are described in detail in the Supplementary Methods.

### 2.3 LLM selection and response generation

Five LLMs were evaluated: two proprietary reasoning-optimized models (O1, O3-mini-high), one proprietary general-purpose model (GPT-4), one biomedical-tuned model (Llama3-OpenBioLLM-70B), and one open-source reasoning-optimized model (DeepSeek-R1). These models were selected to reflect diversity in architecture and training strategy, spanning general-purpose, biomedical-specialized, and reasoning-augmented approaches. Selection was guided by clinical relevance, prior benchmark performance, and recent advances in multi-step reasoning and medical natural language processing (2, 11–13). For each scenario, responses were generated using a multi-agent prompting strategy (14). Specifically, three independent seed responses were first generated using separate chat sessions to ensure output diversity. The same model was then prompted with a master query that included all three seed responses and instructed the model to synthesize them into a single consolidated answer. This two-stage process was applied uniformly across all five models. The exact seed and master prompt templates are provided in Supplementary Table S3, and detailed model specifications, including version identifiers, access methods, and deployment parameters, are provided in Supplementary Table S4.

### 2.4 Evaluation process and statistical analysis

In Round 1, evaluators independently reviewed anonymized model responses without access to reference answers. In Round 2, they were shown the suggested answers and invited to reconsider their initial ratings. Revisions were optional, and evaluators were explicitly instructed to preserve independent clinical judgment. A custom web-based application was used to present randomized, blinded responses and streamline scoring (Supplementary Figure S1). Statistical analyses included Fleiss' kappa, Wilcoxon, McNemar, Kendall's W, and Student t-tests as appropriate.

To quantify patterns of human–LLM collaboration, we defined three trust calibration metrics derived from rating shifts between evaluation rounds. The overacceptance rate was defined as the proportion of individual binary ratings revised from affirmative (“Yes”) to negative (“No”) in the second round, quantifying how often evaluators initially endorsed LLM outputs that were subsequently identified as flawed upon reviewing the reference answers. The underrecognition rate was defined as the proportion of ratings revised from negative to affirmative, quantifying how often evaluators initially dismissed LLM outputs that were later deemed clinically appropriate. The net trust calibration gap was calculated as the overacceptance rate minus the underrecognition rate; positive values indicate a predominance of overacceptance. These metrics were computed across all binary ratings (5 evaluators × 5 models × 15 scenarios × 10 dimensions = 3,750 total ratings) and stratified by model, evaluation dimension, and clinical scenario.

Additional methodological and technical details are provided in Supplementary Methods.

## 3 Results

### 3.1 Inter-evaluator consistency

Fleiss' kappa statistics showed limited initial agreement among the five evaluators (*κ* = 0.0834; 95% CI: 0.0545–0.1122; *p* < 0.0001). Agreement improved after the second-round review incorporating suggested answers (*κ* = 0.1298; 95% CI: 0.0981–0.1615; *p* < 0.0001). These findings indicate that reference answers helped recalibrate judgment and enhance alignment without eliminating independent reasoning. The relatively low initial agreement is expected in complex open-ended clinical reasoning evaluations, where multiple management strategies may be considered clinically acceptable.

### 3.2 Overall model performance

**Figure 2:**
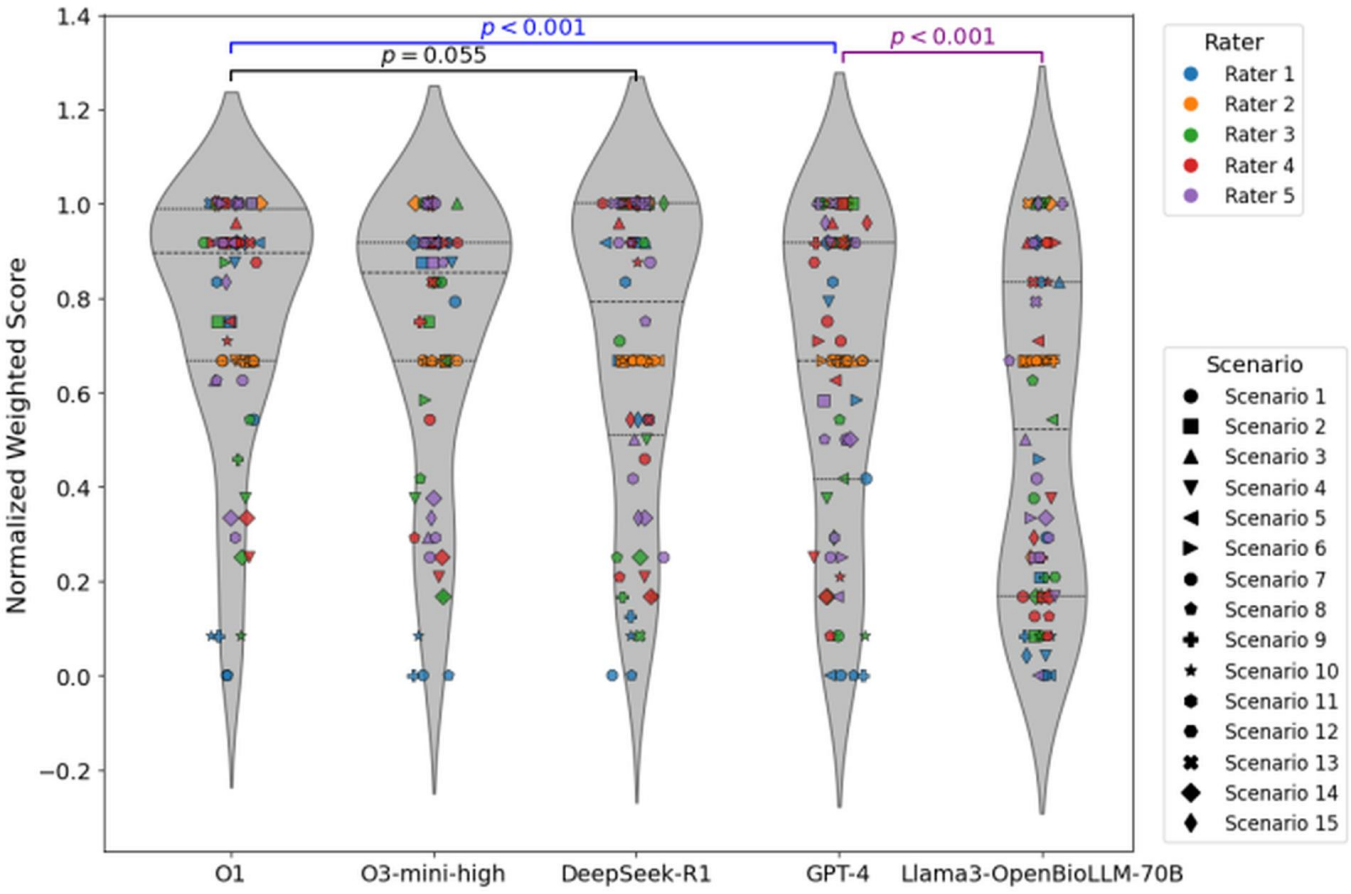
Second-Round normalized scores for five LLMs across 15 cardiac surgery scenarios. This violin plot displays the distribution of normalized weighted scores (range: 0–1) assigned to five LLMs across 15 complex cardiac surgery scenarios. Each violin represents aggregated scores for one LLM. Colored markers represent individual expert evaluators (*n* = 5), while marker shapes denote specific clinical scenarios (*n* = 15). Dashed lines indicate medians and interquartile ranges. Normalization was performed by dividing each raw score (0–24 scale) by the maximum possible score of 24, allowing for standardized comparison across models.

Model performance was assessed using second-round ratings, which integrated evaluator judgment with reference answers to provide a more robust evaluation. As shown in Figure 2, each model was scored using a 24-point weighted framework and normalized to a 0–1 scale for comparability. Median normalized scores [25th–75th percentile] were highest for O1 [0.896 (0.667–0.990)], followed by O3-mini-high [0.854 (0.667–0.917)], DeepSeek-R1 [0.792 (0.510–1.000)], GPT-4 [0.667 (0.417–0.917)], and Llama3-OpenBioLLM-70B [0.521 (0.167–0.833)].

To evaluate the effect of reasoning optimization, O1 (reasoning-optimized) was compared to GPT-4 (non-reasoning), with O1 scoring significantly higher (Wilcoxon *p* < 0.001; mean difference = +0.106). Among reasoning-enabled models, O1 (proprietary) outperformed DeepSeek-R1 (open-source), though the difference was marginal (Wilcoxon *p* = 0.055; mean difference = +0.049). Among non-reasoning models, GPT-4 significantly outperformed Llama3-OpenBioLLM-70B (Wilcoxon *p* < 0.001; mean difference = +0.140).

Full score breakdowns across evaluation dimensions and clinical scenarios for both rounds are provided in Supplementary Results.

### 3.3 LLM performance across evaluation dimensions and clinical scenarios

**Figure 3:**
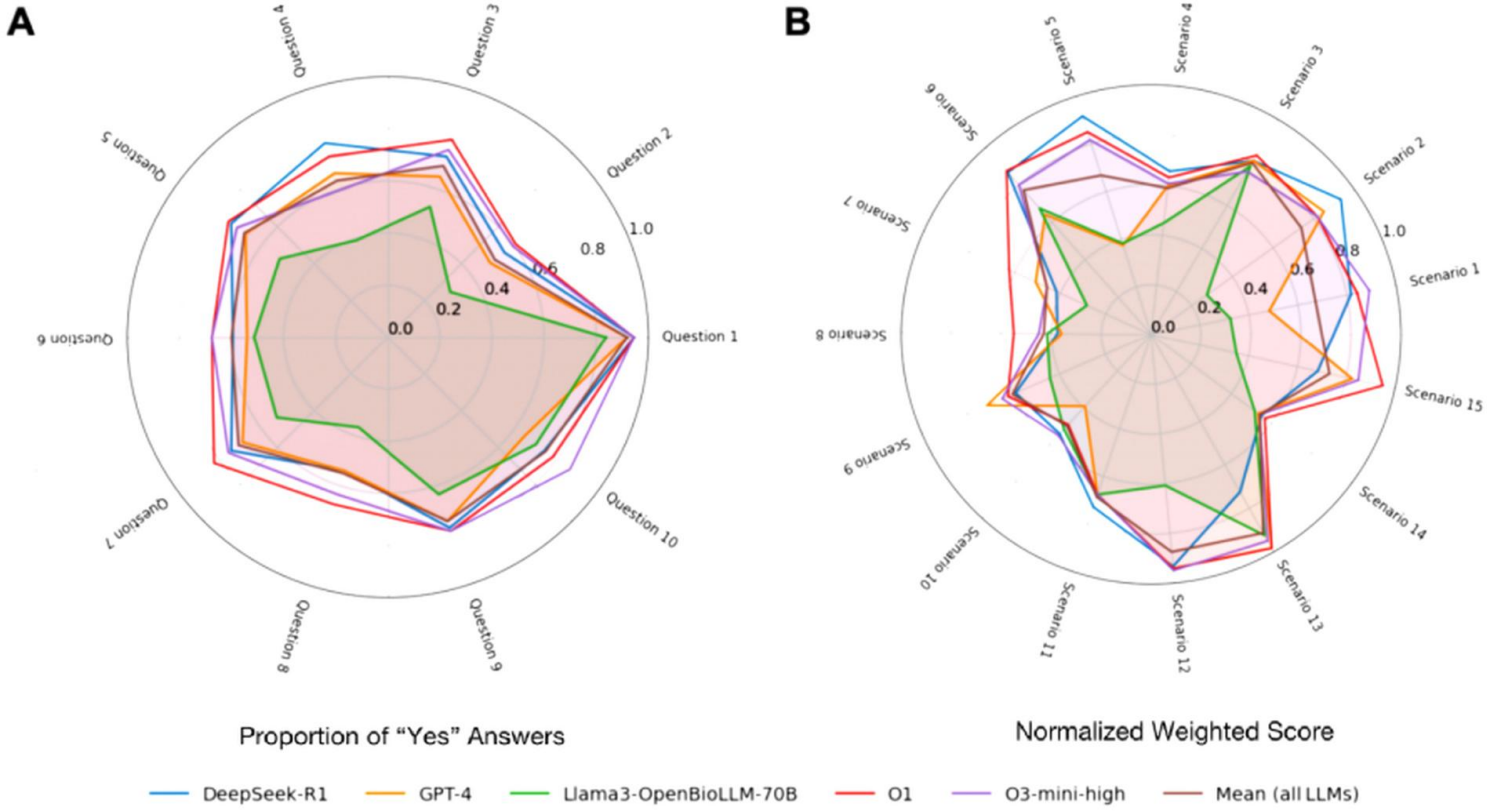
Second-Round evaluation of LLM performance across evaluation dimensions and clinical scenarios. **(A)**. LLM Performance Across Ten Clinical Evaluation Dimensions. Radar plot displaying the average proportion of “Yes” ratings (range: 0–1) achieved by each LLM across ten evaluation dimensions. Each axis represents one binary-scored clinical question used in the evaluation framework (Q1–Q10). Colored lines denote model-specific means and the average performance across all models. Larger values indicate stronger performance on the corresponding dimension. **(B)**. Normalized LLM Performance Across 15 Cardiac Surgery Scenarios. Radar plot showing normalized weighted scores (range: 0–1) for five LLMs across 15 clinical scenarios. Each colored line represents an individual LLM and the mean score across all models. The plot illustrates model-specific variability and comparative performance across scenarios.

Among the ten evaluation dimensions, scenario comprehension (Q1) achieved the highest mean score (0.920). In contrast, several critical domains revealed significant weaknesses. Patient safety (Q2) had the lowest average score (0.507); even the best-performing model, O1, reached only 0.600, while Llama3-OpenBioLLM-70B scored 0.307. Clinical efficiency (Q6) and hallucination avoidance (Q8) also showed relatively poor performance (mean scores: 0.597 and 0.549, respectively), with O1 scoring 0.667 on both. O1 ranked first in 8 of the 10 dimensions, achieving the highest overall performance (Figure 3A). Despite variation in absolute scores, evaluation dimension-level rankings were consistent across models (Kendall's *W* = 0.789, *p* < 0.001), suggesting shared patterns of strength and weakness.

Model performance also varied across the 15 clinical scenarios. O1 ranked highest overall and achieved the top score in 7 scenarios, reflecting strong stability across task types. O3-mini-high and DeepSeek-R1 also performed well, though with slightly greater variability. In contrast, Llama3-OpenBioLLM-70B consistently underperformed. Scenarios 12 and 13 yielded the highest average scores across models (0.875 and 0.913, respectively), while Scenarios 7 and 8 had the lowest (0.458 and 0.430, respectively) (Figure 3B). Scenario-level rankings were broadly consistent (Kendall's *W* = 0.323, *p* = 0.0007), underscoring the influence of scenario complexity and supporting the robustness of model performance patterns.

### 3.4 Trust calibration patterns in human–LLM collaboration

**Figure 4:**
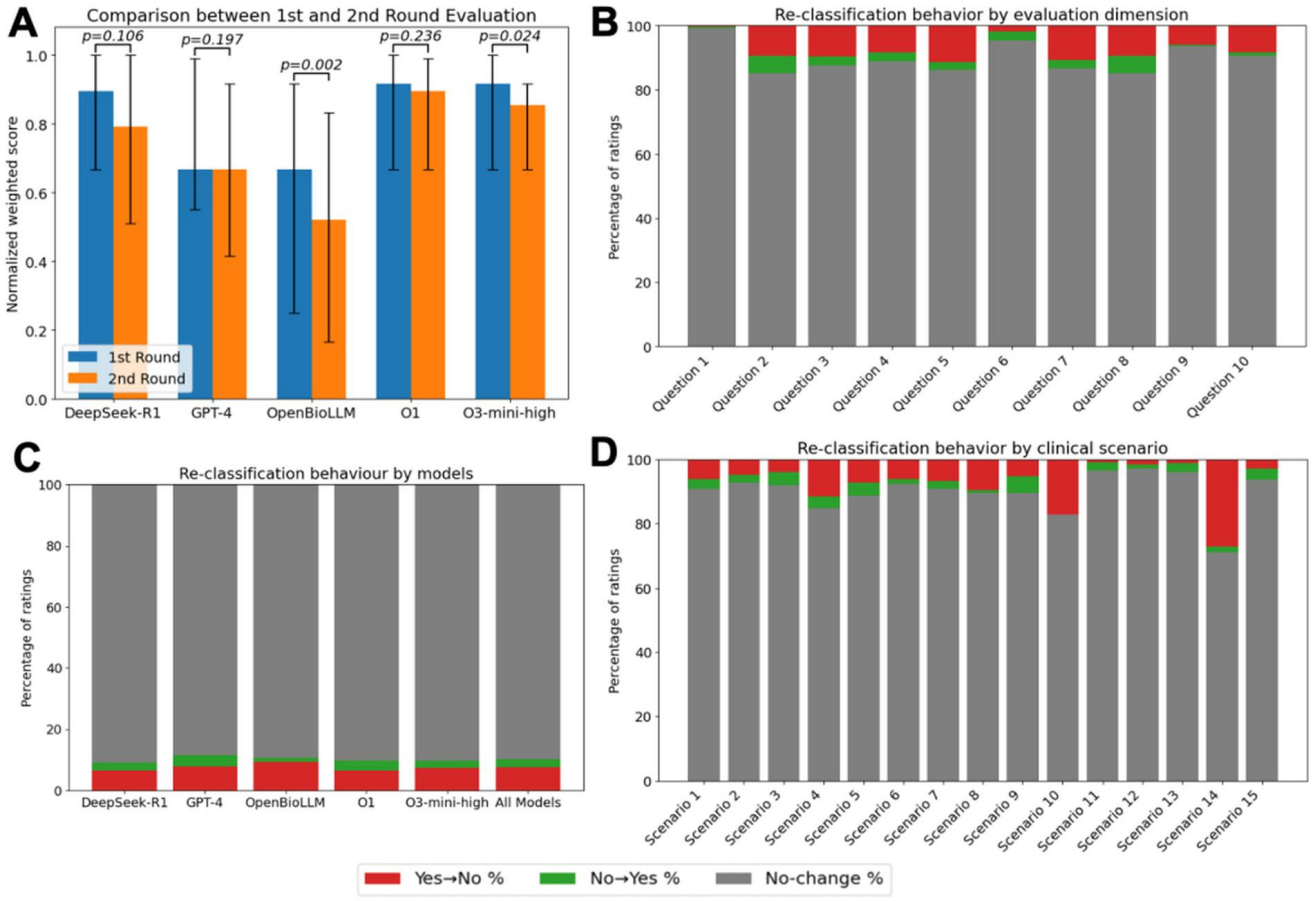
Trust calibration patterns in Two-phase human–LLM collaboration evaluation. **(A)**. First-Round vs. Second-Round Mean Scores Across LLMs. Normalized weighted scores (0–1 scale) before and after evaluators viewed suggested answers; **(B)**. Rating Changes by Evaluation Dimension. Proportions of unchanged, affirmative-to-negative, and negative-to-affirmative ratings across all ten evaluation dimensions; **(C)**. Rating Changes by LLM. Proportions of unchanged, affirmative-to-negative, and negative-to-affirmative ratings for each LLM; **(D)**. Rating Changes by Clinical Scenario. Proportions of unchanged, affirmative-to-negative, and negative-to-affirmative ratings across 15 scenarios. “Yes→No” and “No→Yes” indicate affirmative-to-negative and negative-to-affirmative rating changes, respectively.

To quantify human–LLM collaboration, we analyzed trust calibration patterns based on rating shifts between rounds. The overall overacceptance rate (affirmative-to-negative revisions) was 7.57%, whereas the underrecognition rate (negative-to-affirmative revisions) was 2.59%, yielding a net trust calibration gap of +4.98% (McNemar's test, *p* < 0.001). This confirms that overacceptance—experts initially endorsing flawed LLM reasoning that appeared clinically sound—was the dominant mode of collaboration failure. As shown in Figure 4A, four of the five LLMs showed a decline in normalized scores in the second round. The largest decline occurred with Llama3-OpenBioLLM-70B (−0.146, *p* = 0.002), followed by O3-mini-high (−0.063, *p* = 0.024). DeepSeek-R1 also declined (−0.104), though the difference was not statistically significant, likely due to greater scoring variability. GPT-4's average score remained stable, reflecting offsetting upward and downward revisions rather than true collaboration alignment.

In total, 10.16% of second-round ratings were revised from their initial values—7.57% from affirmative to negative and 2.59% from negative to affirmative—indicating a predominance of downward revisions (*p* < 0.001, McNemar's test). Across all evaluation dimensions, the highest overall change rates occurred in patient safety (Q2) and hallucination avoidance (Q8), both at 14.86%. In contrast, scenario comprehension (Q1) showed the lowest change rate (0.81%). Notably, among the five most heavily weighted dimensions (weight ×3), 10.16% of ratings were revised from affirmative to negative: patient safety (Q2, 9.46%), evidence-based reasoning (Q3, 9.73%), patient benefit (Q5, 11.35%), helping decision-making (Q7, 10.81%), and hallucination avoidance (Q8, 9.46%). In contrast, only 3.68% of ratings for these same dimensions were upgraded, underscoring a consistent trend toward downgrading in clinically critical areas (Figure 4B).

The trust calibration gap was most pronounced in these safety-critical domains: among the five highest-weighted dimensions (Q2, Q3, Q5, Q7, Q8), the overacceptance rate (10.16%) was nearly three times the underrecognition rate (3.68%), yielding a net trust calibration gap of +6.48%. This indicates that expert trust miscalibration was concentrated in precisely the dimensions where errors carry the greatest clinical consequence—a finding consistent with automation bias, whereby plausible and fluently structured outputs reduce the likelihood of critical human scrutiny. At the model level, Llama3-OpenBioLLM-70B exhibited the highest overacceptance rate (9.46%) and the lowest underrecognition rate (1.22%), indicating it most frequently produced misleading outputs that escaped expert detection. In contrast, O1 had the lowest overacceptance rate (6.49%) and a relatively higher underrecognition rate (3.38%), suggesting its outputs were more often appropriately calibrated with evaluator judgment.

As shown in Figure 4C, the proportion and direction of rating changes varied across LLMs. GPT-4 had the highest overall change rate (11.49%), followed by Llama3-OpenBioLLM-70B (10.68%), O1 (9.86%), O3-mini-high (9.73%), and DeepSeek-R1 (9.05%). Across models, affirmative-to-negative changes consistently outnumbered negative-to-affirmative ones. Llama3-OpenBioLLM-70B had the highest rate of downward changes (9.46%); in contrast, O1 had the lowest (6.49%). For upward changes, GPT-4 led (3.65%), followed by O1 (3.38%) and DeepSeek-R1 (2.43%), while Llama3-OpenBioLLM-70B again ranked lowest (1.22%). These patterns highlight differences in human–LLM collaboration: some models (e.g., Llama3-OpenBioLLM-70B) more often elicited overacceptance of flawed outputs, and others provided more valuable information that was initially underrecognized (e.g., GPT-4).

As shown in Figure 4D, 12 of the 15 clinical scenarios exhibited downward-skewed rating changes, indicating that LLM responses were frequently overestimated on first review. Scenarios 14 and 10 showed the highest proportions of affirmative-to-negative revisions (27.2% and 17.2%). In contrast, only three scenarios—11, 13, and 15—had more upward than downward changes. Minimal overall change (<5%) was observed in Scenarios 11, 12, and 13. These findings underscore the limitations of first-pass assessments in open-ended, free-text evaluations and demonstrate how scenario complexity shapes both the difficulty of judgment and the pattern of human–LLM collaboration imbalance.

Full visualizations of first-round performance are provided in Supplementary Results.

## 4 Discussion

### 4.1 Overview of key findings and implications

Direct evaluation of LLMs in high-stakes surgical settings remains impractical under patient safety and workflow constraints. As prior studies suggest, expert-designed clinical scenarios offer a validated approach to simulate real-world complexity while avoiding model pre-exposure (15, 16). Building on this, we developed diverse, high-fidelity cardiac surgery cases to assess LLM competencies—including multidisciplinary reasoning, complex pathophysiological interpretation, high-risk decision-making, and advanced medical calculations. Compared to multiple-choice formats that often oversimplify reasoning (17), these free-text tasks and model outputs better reflect the cognitive demands of real-world practice. We also introduced a two-phase evaluation using a structured ten-dimensional framework. The first round reflected real-world use, with clinicians judging LLM responses based on initial impressions. The second round, after reviewing reference answers, enabled a more rigorous assessment and insight into human–LLM collaboration.

Our results highlight that strong performance by LLMs on standardized exams does not ensure safe or reliable application in complex, high-stakes clinical decision-making. While reasoning-optimized models performed better overall and open-source models showed progress toward proprietary systems, even the top-performing models exhibited critical limitations. Notably, even experienced surgeons often failed to recognize LLM errors at first glance—a finding that reflects not only the potential for LLMs to produce plausible yet misleading outputs, but also the limitations of unaided, first-pass evaluation. This highlights a predominant pattern of overacceptance in human–LLM collaboration when LLMs are used in cardiac surgery, which may further increase the clinical risks.

### 4.2 Reasoning-optimized models perform better, but all fall short on safety and reliability

Among the five models evaluated, O1 performed best overall, followed by O3-mini-high and DeepSeek-R1, while GPT-4 and Llama3-OpenBioLLM-70B ranked lower. These differences reflect variations in architecture, reasoning optimization, and training strategies. Reasoning-optimized models (O1, O3-mini-high, DeepSeek-R1) showed more stable performance, likely due to chain-of-thought prompting and supervised multi-step pathways. These promote coherent logic, reduce overconfident errors, and improve alignment with task demands, particularly in domains requiring sequential reasoning. This aligns with recent findings that structured prompting and multi-step reasoning strategies enhance probabilistic reliability, support faithful reasoning traces, and improve LLM performance (18). GPT-4, though widely used, is not reasoning-optimized. Its responses were concise and well-structured, but often too generic for complex surgical contexts. Llama3-OpenBioLLM-70B, despite biomedical tuning, underperformed; its outputs often lacked accuracy and task relevance, suggesting domain exposure alone is insufficient without embedded reasoning. Consistent with prior work on guideline adherence across specialties (19), our findings showed that reasoning-optimized models outperformed non-reasoning models regardless of licensing status. Among reasoning-enabled architectures, the proprietary O1 marginally outperformed the open-source DeepSeek-R1, suggesting that the performance gap between proprietary and open-source systems is narrowing when both incorporate inference-time reasoning capabilities. These systems may soon offer viable, lower-cost alternatives with potential advantages in customization and data privacy (20). Notably, even O1—the top performer—showed clear deficits in core dimensions like patient safety and hallucination avoidance, indicating insufficient reliability for independent use in complex surgical decision-making.

To illustrate these safety failures, we highlight two examples from the scenarios with the highest overacceptance rates. In Scenario 14, which required selecting a mitral annuloplasty ring size from echocardiographic data, four of five models recommended 30–32 mm, whereas the reference answer indicated 36–38 mm. A 6–8 mm undersizing error risks systolic anterior motion, left ventricular outflow tract obstruction, or persistent mitral regurgitation. The models supported their recommendations with stepwise sizing logic and specific anatomical references, creating an appearance of methodological rigor that masked the incorrect conclusion. In Scenario 10, involving hemodynamic deterioration after a Fontan procedure, the reference answer identified preload insufficiency as the primary cause and recommended fluid resuscitation with positive end-expiratory pressure reduction to support the passive Fontan circulation. However, GPT-4 recommended diuretic therapy, a directly contradictory intervention that would further deplete preload, risking circulatory collapse. This dangerous recommendation was embedded within an otherwise comprehensive and largely correct clinical analysis, making it difficult to isolate during rapid review. These examples reflect two primary mechanisms by which high-risk hallucinations evade expert detection: (1) a systematically flawed quantitative reasoning chain was presented with sufficient methodological detail to appear credible, and (2) isolated critical errors concealed within a largely accurate and comprehensive clinical analysis.

### 4.3 LLMs struggle beyond structured clinical knowledge

We next examined LLM performance across clinical scenarios, which spanned a wide range of cardiac surgical complexity. A *post-hoc* complexity analysis (Supplementary Table S6), in which the three scenario designers independently rated each case across five clinical dimensions, confirmed that performance was broadly associated with scenario complexity. The highest-scoring scenarios (Scenarios 12 and 13; complexity scores 8.0 and 10.0) involved linear reasoning and structured clinical cues, such as managing postoperative respiratory decline or reversing intraoperative right ventricular compression during off-pump coronary bypass surgery. Both followed well-mapped pathophysiological processes and textbook-based management strategies, closely aligned with structured knowledge that LLMs are trained on. In contrast, the lowest-scoring scenarios (Scenarios 7, 8, and 10; complexity scores 14.0–14.3) involved more intricate pathophysiological processes, such as recognizing postoperative restrictive right ventricular physiology in Tetralogy of Fallot, managing pulmonary-to-systemic blood flow imbalance in single-ventricle anatomy, or addressing hemodynamic deterioration after a Fontan procedure. These tasks demanded integration of dynamic variables, longitudinal reasoning, and highly specialized congenital cardiac expertise—areas where LLMs struggled, likely due to limited subspecialty representation in training data and real-time reasoning constraints. Notably, complexity and LLM performance did not always align: Scenario 14 (mitral annuloplasty ring sizing) had a moderate complexity score (9.3) yet yielded low LLM performance (0.542), suggesting that certain task types—particularly those requiring precise quantitative reasoning with device-specific parameters—pose challenges to LLMs that are not fully captured by clinical complexity alone. Overall, LLMs generalized more effectively in guideline-driven, structured scenarios than in abstract or variable-dense clinical contexts. This aligns with findings from general medical question answering settings, where high-performing LLMs faltered when reasoning beyond structured knowledge (15).

### 4.4 Two-round evaluation reveals imbalances in human–LLM collaboration

The integration of artificial intelligence into cardiac surgery is expected to shift surgeons from direct operators to supervisors (21) —a transformation that raises the critical, yet understudied, question of whether surgeons can effectively evaluate and utilize LLM outputs. To investigate human–LLM collaboration, we implemented a two-phase evaluation framework that enabled analysis of rating shifts between rounds. Three patterns emerged: First, scores improved in the second round, reflecting underrecognition—clinically sound responses were initially undervalued, indicating some opportunities to leverage the model's potential were missed; Second, scores declined, reflecting overacceptance—plausible but flawed outputs escaped first-pass evaluation, leading to inappropriate endorsement; Third, scores remained unchanged, which may suggest appropriate evaluation and optimal use; it may also result from offsetting effects, where underrecognition and overacceptance cancel each other out. These patterns reveal distinct modes of human–LLM collaboration and underscore the need to evaluate not only model output accuracy, but also how outputs shape clinician judgment.

Our results showed that four LLMs experienced a decline in scores during the second round, consistent with the overacceptance pattern of human–LLM collaboration. This decline was statistically significant for both Llama3-OpenBioLLM-70B and O3-mini-high, suggesting these models more often produced misleading outputs that went unchallenged. In contrast, GPT-4 did not show a net decline, but this reflected offsetting effects: many downward revisions were balanced by upward adjustments, particularly in high-weighted items. Therefore, it does not represent truly aligned human–LLM collaboration. A recent study showed that when physicians collaborated with LLMs, diagnostic accuracy did not improve, and in some cases was lower than that of the LLMs alone, due to clinicians underutilizing correct model suggestions (6). In our study, the opposite imbalance—overacceptance—emerged as the dominant pattern. Evaluators frequently assigned higher scores on initial review, indicating that even experienced clinicians may overlook subtle but clinically significant errors, especially under time pressure and cognitive load in complex, information-dense decision-making settings like cardiac surgery. This overacceptance may reflect a broader cognitive mechanism: when LLM outputs appear fluent and well-structured, users are prone to shift from active reasoning into passive exploitation, prematurely accepting suggestions without critical evaluation (22).

Quantitatively, the net trust calibration gap in our study was +4.98% overall, indicating systematic over-trust in LLM outputs across all models. This gap widened substantially in safety-critical domains: among the five highest-weighted evaluation dimensions, the overacceptance rate (10.16%) was nearly three times the underrecognition rate (3.68%), yielding a net gap of +6.48%. These findings indicate that the fluency and structural coherence of LLM-generated outputs were particularly effective at suppressing critical evaluation in the domains most relevant to patient safety—a pattern consistent with established models of automation bias in clinical decision support systems, where confident, well-formatted outputs reduce human vigilance even among experienced practitioners.

From an evaluation dimension perspective, the highest rates of affirmative-to-negative changes occurred in two high-weighted domains: patient benefit and helping decision-making. This points to a recurrent failure mode: LLM outputs often appeared confident and well-reasoned on first impression, but were later reassessed as lacking clinical depth or actionable value. The polished language encouraged overacceptance by creating an illusion of correctness, while masking gaps in specificity or utility. The clinical context also shaped patterns of human–LLM collaboration. Scenarios with high downward revisions typically involved structured reasoning tasks, such as mitral annuloplasty ring size prediction using echocardiography data or hemodynamic reasoning using physiologic indicators, where LLMs mimicked expert logic in form but embedded subtle clinical errors. In contrast, upward rating shifts were more common in interpretive tasks, such as selecting appropriate interventions for hypotension. These tasks lacked obvious red flags and involved several overlapping or subtle clinical cues, some of which may have been underweighted by evaluators during rapid first-pass review. The structured format of LLM responses often captured a broad range of underappreciated details, like elevated lactate or low urine output, prompting reappraisal. Together, these findings show that overacceptance and underrecognition varied not only across evaluation dimensions but also depending on the clinical scenario.

### 4.5 Study limitations

First, the study did not include imaging inputs, such as echocardiographic views or intraoperative visuals, that are often critical for surgical decision-making. This limitation was partially mitigated by structured clinical summaries and imaging reports, which reflect how such information is typically conveyed in real-world workflows. As noted in prior work (16), LLMs benefit from rich textual context, whereas their capacity for raw image interpretation remains limited. Future studies should incorporate multimodal data and larger scenario sets to better reflect real-world clinical practice.

Second, inter-evaluator agreement on individual items was modest. This is expected, as variation in clinical judgment is common in real-world cardiac surgery, particularly when scoring nuanced, free-text responses. To reduce subjectivity, we used a ten-dimensional binary framework, a two-phase blinded review, and multiple independent evaluators, allowing individual judgment while minimizing shared bias. These measures improved reliability, especially in the second round, though variability is inevitable. Notably, evaluators showed strong agreement in broader patterns: they consistently distinguished higher-from lower-performing models and showed stable trends across evaluation dimensions and scenarios. Recent work has adopted conceptually similar strategies—structured simulation with expert adjudication—to address subjectivity in free-text LLM evaluation (17).

Third, our study design evaluated how experts assessed LLM-generated outputs rather than measuring whether AI assistance improved or hindered independent diagnostic accuracy. Because evaluators reviewed LLM responses from the outset rather than first generating their own clinical judgments without AI input, we cannot directly quantify the net impact of AI-generated rationales on the experts' own decision-making accuracy. Nevertheless, the trust calibration metrics introduced in this study, overacceptance and underrecognition rates, capture a complementary dimension of collaboration risk that accuracy-comparison designs do not address. Future studies incorporating a pre-AI independent assessment phase would enable direct measurement of the impact of LLM-generated reasoning on diagnostic accuracy.

Fourth, cross-model comparisons in this study are subject to architectural and methodological asymmetries. Llama3-OpenBioLLM-70B, a 70-billion-parameter dense model, was evaluated alongside proprietary models that are likely substantially larger, confounding the effects of domain-specific fine-tuning with raw model capacity. Additionally, the uniform application of identical prompting templates across architecturally diverse models may have differentially advantaged reasoning-optimized architectures, which benefit from internal chain-of-thought mechanisms and inference-time computation that standard models lack. While our use of chat-style web interfaces with default settings reflects typical clinician usage, it precludes fine-grained parameter matching across models. These asymmetries should be considered when interpreting cross-model performance differences.

## 5 Conclusions

Reasoning-optimized LLMs outperformed their peers in complex cardiac surgery scenarios, yet still failed at unacceptable rates in critical safety-related dimensions. While LLMs handled linear, guideline-based tasks well, they struggled with scenarios requiring longitudinal, uncertainty-tolerant reasoning. Crucially, the greatest risk may lie not in what models get wrong, but in how plausibly they do so. Trust calibration analysis revealed that overacceptance—rather than underrecognition—was the dominant mode of human–AI collaboration failure, with miscalibration rates nearly tripling in the five highest-weighted, safety-critical evaluation dimensions. Future LLMs must be evaluated not only for output accuracy but for how they influence human–LLM collaboration, since clinical impact depends as much on interaction as on information. Safeguards such as secondary review, task-specific restrictions, interface-level strategies (e.g., uncertainty tagging, factuality scoring), and retrieval-augmented generation approaches may improve human–LLM collaboration by enhancing factual accuracy and enabling transparent verification (23, 24).

## Data Availability

The datasets generated and analyzed in this study, including the clinical scenarios and model responses, are included in the Supplementary Material. Further inquiries can be directed to the corresponding author/s.
