# Supplementary material for: Blinded two-phase evaluation of large language models in complex cardiac surgery: task-specific performance and human-AI collaboration
Source: Front Digit Health. 2026 May 29;8:1769467. doi: 10.3389/fdgth.2026.1769467 (PMC13260534; doi:10.3389/fdgth.2026.1769467)
Supplement: Supplementary file 1 [file Table1.docx]

Supplementary results

This section presents first-round results, based on initial evaluator ratings without access to reference answers. Median normalized scores [25th–75th percentile] were highest for O1 and O3-mini-high (0.917 [0.667–1.000]), followed by DeepSeek-R1 (0.896 [0.667–1.000]), GPT-4 (0.667 [0.552–0.990]), and Llama3-OpenBioLLM-70B (0.667 [0.250–0.917]).

As shown in Supplementary Figure S2A, O1 (reasoning-optimized) significantly outperformed GPT-4 (non-reasoning) (Wilcoxon *p* < 0.001; mean difference = +0.097). Among reasoning-enabled models, O1 (proprietary) slightly outperformed DeepSeek-R1 (open-source) (Wilcoxon *p* = 0.281; mean difference = +0.035). Among non-reasoning models, GPT-4 significantly outperformed Llama3-OpenBioLLM-70B (Wilcoxon *p* = 0.029; mean difference = +0.092).

As shown in Supplementary Figure S2B, across the ten evaluation dimensions, scenario comprehension (Q1) yielded the highest average score (0.916). In contrast, patient safety (Q2) scored lowest (0.549), even for the top model, O1 (0.649); Llama3-OpenBioLLM-70B scored 0.405. Clinical efficiency (Q6) and hallucination avoidance (Q8) also showed weak performance (mean scores of 0.592 and 0.592, respectively). O1 ranked first in 7 out of 10 dimensions. Despite variation in absolute scores, dimension-level rankings were consistent across models (Kendall’s W = 0.789, *p* < 0.001), indicating shared strengths and limitations.

As shown in Supplementary Figure S2C, performance also varied across clinical scenarios. O3-mini-high ranked highest overall and achieved the top score in 7 scenarios. O1 and DeepSeek-R1 also performed well. Llama3-OpenBioLLM-70B consistently ranked lowest. Scenarios 12 and 13 yielded the highest average scores (0.875 and 0.893), while Scenarios 7 and 8 scored lowest (0.503 and 0.538). Scenario-level rankings showed moderate consistency across models (Kendall’s W = 0.236, *p* = 0.007), highlighting the role of task complexity in shaping model performance.

The Supplementary Figure S3 displays four heatmaps comparing the performance of five LLMs across two evaluation rounds.
