# Supplementary material for: Blinded two-phase evaluation of large language models in complex cardiac surgery: task-specific performance and human-AI collaboration
Source: Front Digit Health. 2026 May 29;8:1769467. doi: 10.3389/fdgth.2026.1769467 (PMC13260534; doi:10.3389/fdgth.2026.1769467)
Supplement: Supplementary file 6 [file Table6.docx]

Supplementary Methods

# Study Design and Role Assignment

Institutional review board approval and informed consent were waived, as the study involved no patient data or human subjects. This study followed a blinded two-phase evaluation design, and a visual summary of the evaluation structure and research objectives is provided in Figure 1 of the main manuscript. Eight board-certified senior cardiac surgeons participated: three developed clinical scenarios and the evaluation framework; five served as independent evaluators of LLM performance. All participating surgeons were attendings with over 10 years of independent clinical experience in cardiac surgery.

# Clinical Scenarios and Evaluation Framework Design

The three surgeons collaboratively developed 15 high-fidelity clinical scenarios with associated clinical tasks, drafted reference answers for each task, and constructed a ten-dimension framework to evaluate LLM responses. Throughout the process, they discussed and consulted relevant literature and clinical guidelines. Each scenario was designed to reflect authentic clinical encounters, such as bedside discussions, interdisciplinary rounds, or intraoperative decision-making. Clinical tasks were written from the perspective of a consulting physician seeking input from a peer. All materials were expressed in natural medical English to mirror real-world communication. The scenarios encompassed a wide range of cardiac surgical conditions, including congenital heart disease, coronary artery disease, valvular disease, and perioperative arrhythmias. Tasks covered multiple clinical phases and competencies, such as diagnostic reasoning, hemodynamic interpretation, pharmacologic and surgical planning, intraoperative complication management, medical calculations, and real-time therapeutic decision-making (Supplementary Table S1).

The evaluation framework captured 10 clinical domains—including patient safety, evidence-based reasoning, and hallucination avoidance—and was designed to rigorously assess LLM capabilities. Each LLM-generated response to a scenario task was evaluated using 10 binary (Yes/No) items, with weights assigned based on clinical importance (Supplementary Table S2). Weighted item scores were summed per scenario and normalized to a 0–1 scale. Overall model performance was summarized by the median of normalized scenario scores across all raters.

# LLM Selection and Response Generation

We selected five models to provide a representative sample of current LLMs: two proprietary reasoning-optimized models, one proprietary general-purpose model, one open-source biomedical model, and one open-source reasoning-optimized model. All models were accessed between January and February 2025 via official web interfaces or open-source repositories. We included three flagship proprietary models from OpenAI: GPT-4 (general-purpose), and two reasoning-optimized models, O1 and O3-mini-high. GPT-4 was selected due to its widespread clinical use and consistent performance in prior medical benchmarks (1,2). O1 is a large-scale proprietary model trained with extensive chain-of-thought techniques to enhance multi-step reasoning, while O3-mini-high is a smaller, high-performing variant with similar optimization. These two models represent the latest generation of reasoning-augmented LLMs.

We also included two open-source models to represent non-proprietary alternatives. Llama3-OpenBioLLM-70B, developed by Saama AI Labs, is a 70-billion-parameter model specialized for the biomedical domain. It has demonstrated state-of-the-art performance on multiple medical natural language tasks and outperformed GPT-4, Google Gemini, Med-PaLM-1, Med-PaLM-2, and Meditron-70B in recent biomedical benchmarks (3). DeepSeek-R1 is an open-source reasoning-optimized model trained with large-scale reinforcement learning to generate detailed step-by-step solutions. It has achieved reasoning performance comparable to OpenAI’s proprietary models (4) and was selected to represent cutting-edge reasoning capabilities in the open-source domain.

# Response Generation via Multi-Agent Prompting

To improve consistency and reduce variability inherent to LLM-based chatbots, we adopted a multi-agent prompting strategy from Du *et al* (5). For each scenario, we generated K = 3 independent “seed” responses using fresh sessions to ensure output diversity. The same model was then prompted with a “master” query to synthesize these seed responses into a single, consolidated answer. In this step, the model received all three preliminary responses and was instructed to reconcile or integrate them into a coherent output that addressed the clinical task. Prompt templates for both stages are provided in Supplementary Table S3. This two-tiered generation process was applied uniformly to all models. It was designed to improve response completeness and coherence while minimizing evaluator burden by avoiding multiple variant outputs. We acknowledge that this approach offers models an opportunity to internally self-correct or refine their answers, potentially boosting performance. We selected this setup to reflect a best-case usage scenario and to fairly represent the peak capabilities of each model.

# Model Query Implementation

All queries to the models were executed via their chat-style interfaces rather than through programmatic application programming interfaces (APIs), to mimic typical clinician usage. All four cloud-hosted models (GPT-4, O1, O3-mini-high, and DeepSeek-R1) were accessed via Stanford Healthcare's SecureGPT, a secure institutional platform providing chat-style access to supported models. Each query was started in a fresh temporary chat session to avoid context carryover between queries. Llama3-OpenBioLLM-70B was deployed locally via its Hugging Face repository11 on a Google Computing Platform (GCP) instance with four A100 graphics processing units (GPUs); each scenario prompt was submitted independently with the model state reset between queries. All model outputs were collected in text form for subsequent evaluation.

# Evaluation Procedure and Blinded Scoring Process

The remaining five surgeons served as independent evaluators. They were blinded to model identity (responses were anonymized), scenario authorship, and each other’s evaluations. Evaluators were explicitly instructed not to seek outside input during the assessment process. In the first round, each evaluator independently reviewed all 15 scenario responses from each of the five models (total: 75 responses) within a fixed 6–8 hour session. These initial evaluation reflected their unaided clinical judgment based solely on the LLM outputs. In the second round, evaluators were shown the suggested answers for each scenario and invited to reconsider their initial ratings. They were permitted to revise ratings but explicitly instructed to preserve independent clinical reasoning. Changes were optional and alignment with the suggested answers was not required. The second-round evaluations, generated after evaluators reviewed the LLM outputs alongside reference answers, yielded a more robust assessment of model performance. The difference between first- and second-round ratings reflected how evaluator assessments shifted, providing a basis for analyzing patterns of human–LLM collaboration.

We used a custom-built web-based application (Supplementary Figure S1) to standardize the presentation of all scenarios and LLM responses in randomized order and to enable blinded, two-round evaluations—thereby streamlining scoring and minimizing bias.

# Statistical Analysis

The rating scores are represented as medians with interquartile range or by percentages. Given the large number of comparisons across five models, ten evaluation dimensions, and two evaluation rounds, we designated overall model performance (aggregate normalized scores) as the primary outcome of interest. Fleiss’ kappa was applied to analyze the inter-evaluator agreement for both first-round and second-round ratings. Wilcoxon, Kendall's W, Student t-test, and McNemar tests were used to distinguish the rating difference among different LLMs, scenarios, questions, and between the 1st and 2nd round ratings as appropriate. For all statistical analyses, a 2-sided alpha (α)-level of 0.05 was considered statistically significant. All analyses were performed using SciPy version 1.11.2 (Fundamental algorithms for scientific computing in Python, <https://scipy.org/)> (6).

# References – Supplementary Methods Section

1. **Leon M, Ruaengsri C, Pelletier G, Bethencourt D, Shibata M, Flores MQ, et al. Harnessing the power of ChatGPT in cardiovascular medicine: innovations, challenges, and future directions. J Clin Med. (2024) 13(21):6543. doi: 10.3390/jcm13216543**
2. **Rydzewski NR, Dinakaran D, Zhao SG, Ruppin E, Turkbey B, Citrin DE, et al. Comparative evaluation of large language models in clinical oncology. NEJM AI. (2024) 1(5):10.1056/aioa2300151. doi: 10.1056/aioa2300151**
3. **Pal MS, Sankarasubbu M.OpenBioLLMs: advancing open-source large language models for healthcare and life sciences. Hugging Face repository. (2024).**
4. **Guo D, Yang D, Zhang H, Song J, Zhang R, Xu R, et al.
   DeepSeek-R1: incentivizing reasoning capability in large language models via reinforcement learning. arXiv [preprint]. (2025) arXiv:2501.12948.**
5. **Du Y, Li S, Torralba A, Tenenbaum JB, Mordatch I. Improving factuality and reasoning in language models through multi-agent debate. In: Proceedings of the 41st International Conference on Machine Learning. (2023).**
6. **Virtanen P, Gommers R, Oliphant TE, Haberland M, Reddy T, Cournapeau D, et al. SciPy 1.0: fundamental algorithms for scientific computing in Python. Nat Methods. (2020) 17(3):261–272. doi: 10.1038/s41592-019-0686-2**
