# Supplementary material for: Blinded two-phase evaluation of large language models in complex cardiac surgery: task-specific performance and human-AI collaboration
Source: Front Digit Health. 2026 May 29;8:1769467. doi: 10.3389/fdgth.2026.1769467 (PMC13260534; doi:10.3389/fdgth.2026.1769467)
Supplement: Supplementary file 10 [file Datasheet1.pdf]

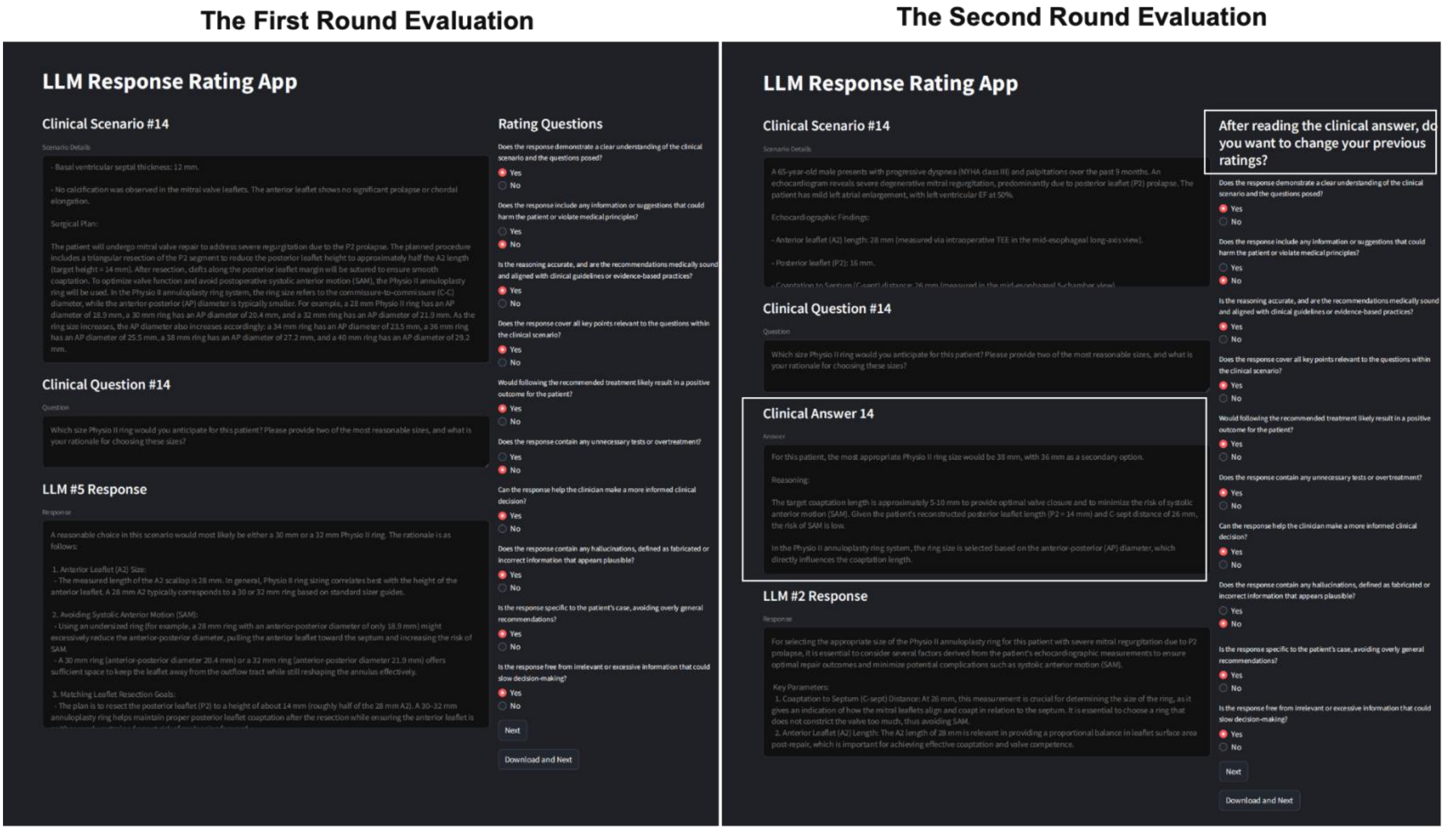

Supplementary Figure S1: Web-based LLM Evaluation APP Interface

The left panel shows the first-round evaluation interface. Evaluators reviewed a clinical scenario, the corresponding clinical question, and a response generated by a LLM, with model identity blinded. On the right, they assessed the LLM's output across ten binary (Yes/No) dimensions reflecting key clinical criteria; The right panel shows the second-round interface for the same scenario. In this phase, a suggested answer was displayed (white box). Evaluators were asked whether they wished to revise their initial ratings and were permitted to update their assessments for each evaluation item.
