# Supplementary material for: Blinded two-phase evaluation of large language models in complex cardiac surgery: task-specific performance and human-AI collaboration
Source: Front Digit Health. 2026 May 29;8:1769467. doi: 10.3389/fdgth.2026.1769467 (PMC13260534; doi:10.3389/fdgth.2026.1769467)
Supplement: Supplementary file 11 [file Datasheet2.pdf]

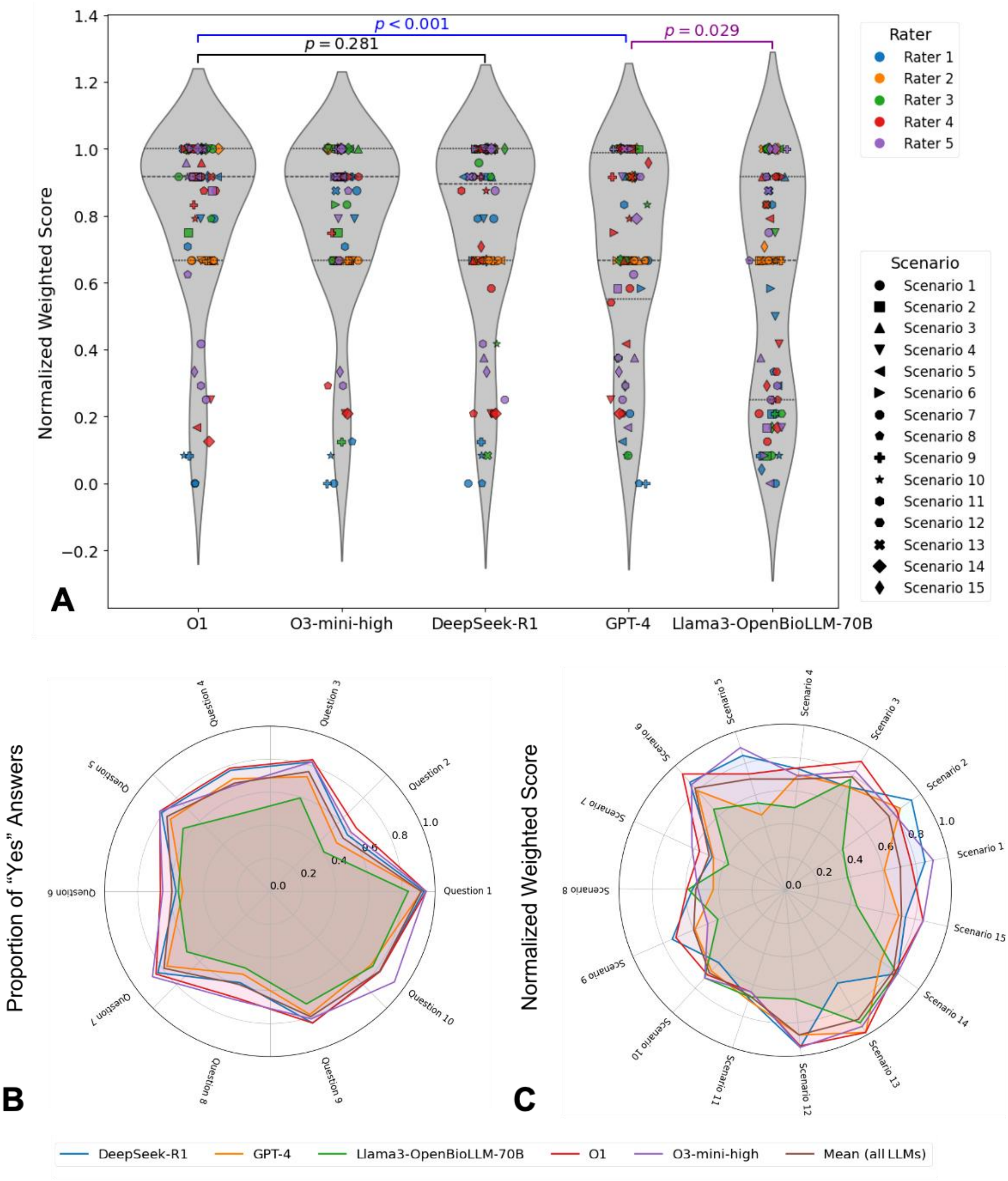

Supplementary Figure S2: First-Round Evaluation Results Across Models, Dimensions, and Scenarios

This figure presents performance results from the first-round evaluation, reflecting initial evaluator assessments of LLM outputs without access to suggested answers, and serving as a baseline for comparison with second-round evaluations. **A.** Normalized Weighted Score Distributions Across Models. Violin plots display the distribution of normalized weighted scores (range: 0–1) for each of the five LLMs across 15 clinical scenarios. Each data point reflects an individual rating; marker shapes denote clinical scenarios, and colors denote evaluators. Dashed lines represent medians and interquartile ranges. Pairwise Wilcoxon p-values are shown for selected comparisons. **B.** Model Performance by Evaluation Dimension. Radar plot summarizing the average proportion of “Yes” ratings (0–1 scale) across ten binary-scored evaluation criteria. Each axis corresponds to one evaluation dimension. Colored lines represent individual LLMs and the overall mean across models. **C.** Model Performance Across Clinical Scenarios.Radar plot displaying the normalized

weighted scores (0–1 scale) achieved by each LLM across 15 cardiac surgery scenarios. Each axis corresponds to a unique clinical case. The variation in patterns highlights model-specific strengths and weaknesses across different task types.
