# Supplementary material for: Blinded two-phase evaluation of large language models in complex cardiac surgery: task-specific performance and human-AI collaboration
Source: Front Digit Health. 2026 May 29;8:1769467. doi: 10.3389/fdgth.2026.1769467 (PMC13260534; doi:10.3389/fdgth.2026.1769467)
Supplement: Supplementary file 12 [file Datasheet3.pdf]

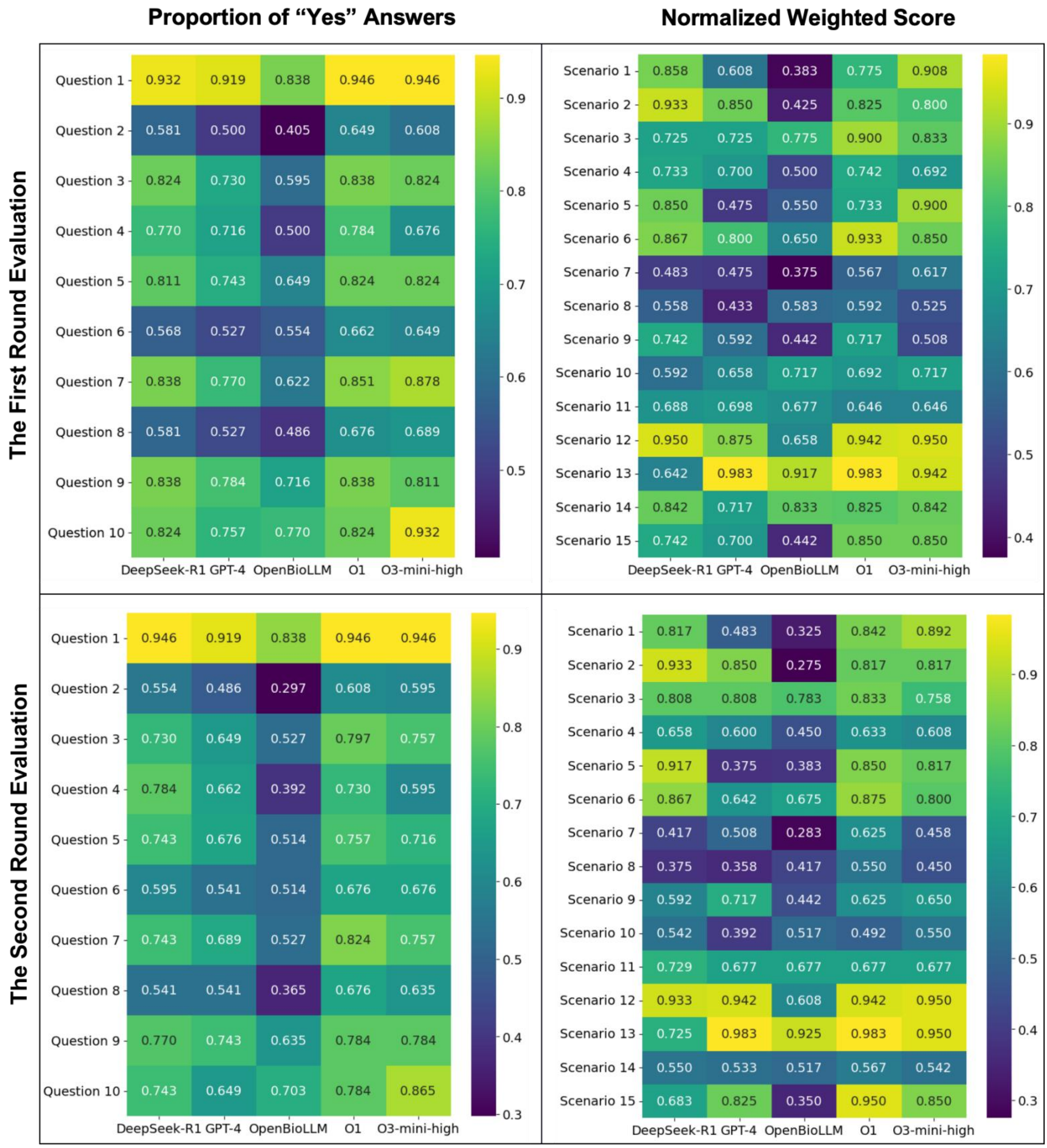

Supplementary Figure S3: First- and Second-Round Evaluation Heatmaps for LLM Performance

The top row presents results from the first-round evaluation, in which expert raters assessed LLM-generated responses to clinical cardiac surgery scenarios without access to reference answers. The bottom row shows second-round results, collected after raters reviewed suggested reference responses and were allowed to revise their initial ratings. In each round, the left heatmap illustrates the proportion of “Yes” responses across 10 binary evaluation dimensions. The right heatmap displays the average normalized weighted score (0–1 scale) for each model across 15 high-fidelity cardiac surgery scenarios.
